# Supplementary material for: Multiple Effects of High Surface Area Hollow Nanospheres Assembled by Nickel Cobaltate Nanosheets on Soluble Lithium Polysulfides
Source: Molecules. 2023 Feb 5;28(4):1539. doi: 10.3390/molecules28041539 (PMC9961496; doi:10.3390/molecules28041539)
Supplement: Supplementary file 1 [file molecules-28-01539-s001.zip › molecules-2181069-supplementary.pdf]

Supporting Information

Multiple Effects of High Surface Area Hollow Nanospheres Assembled by Nickel Cobaltate Nanosheets on Soluble Lithium Polysulfides

Jun Pu, Xiaomei Zhu, Jie Wang, Shaomeng Yu*

Correspondence: jpu@ahnu.edu.cn (J.P.)

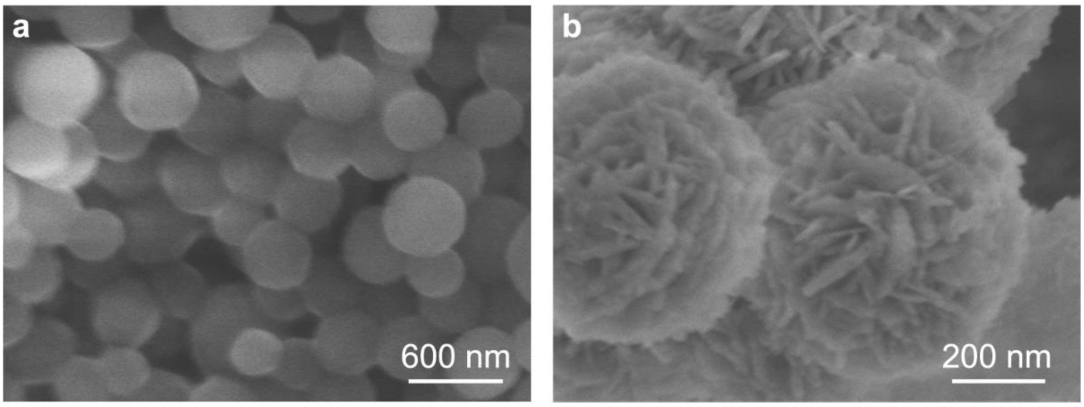

Figure S1. SEM images of (a) carbon sphere templates and (b) Co-Ni precursors.

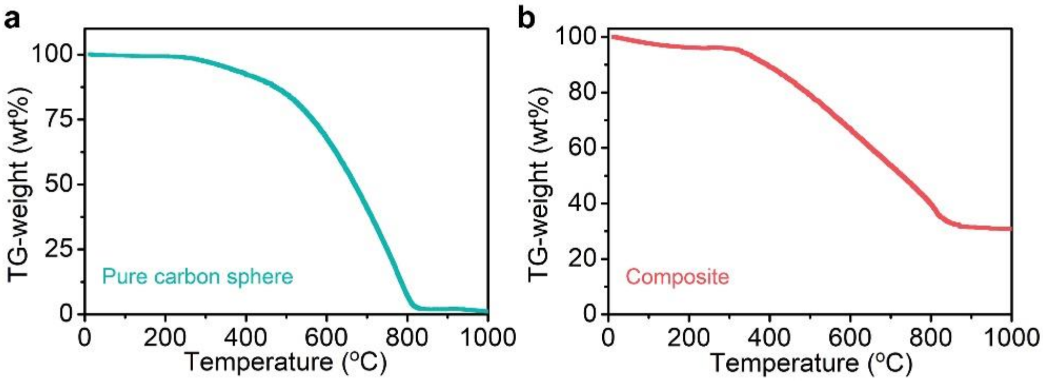

Figure S2. TGA results of (a) pure carbon sphere, and (b) precursor composite.

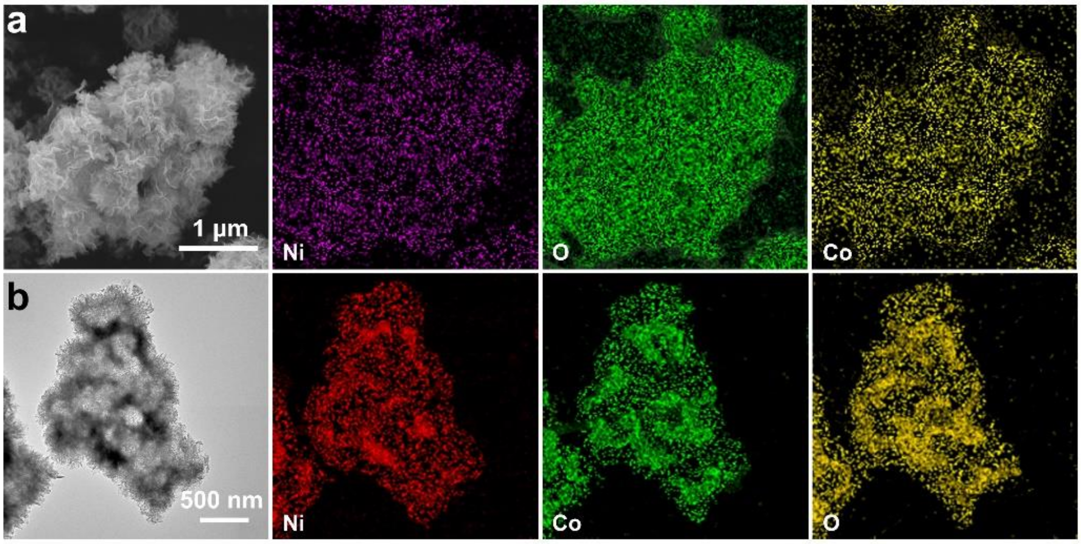

Figure S3. Elemental mapping of $NiCo_2O_4$: (a) SEM, (b) TEM.

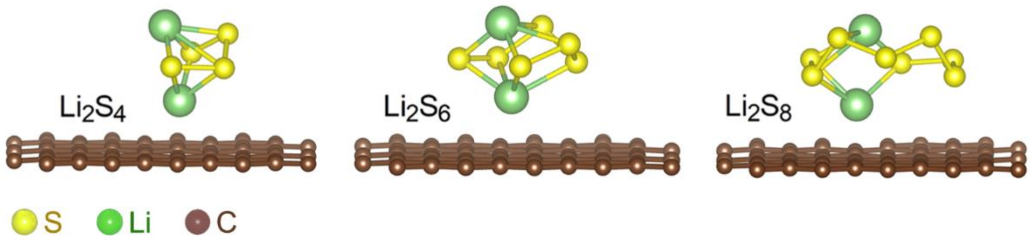

Figure S4. Mechanism diagram of interaction between carbon and LiPSs.

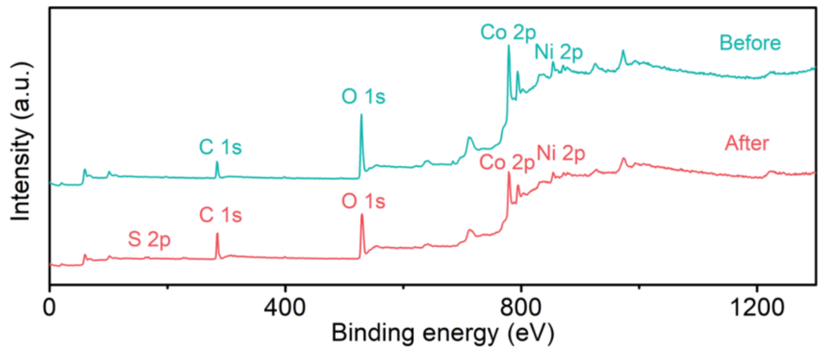

Figure S5. XPS survey spectra of $NiCo_2O_4$ before and after Li_2S_4 adsorption.

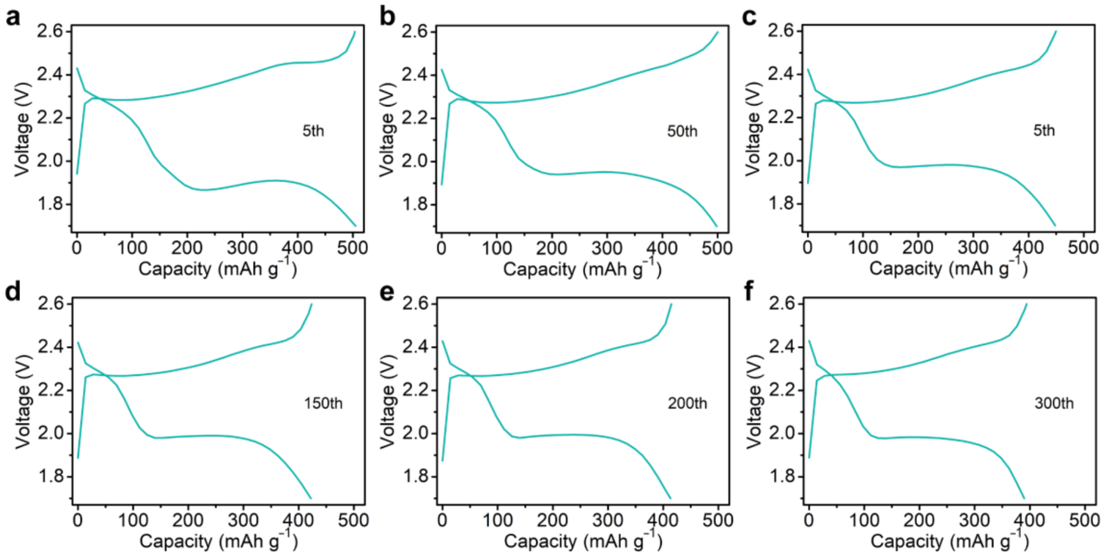

Figure S6. Discharge-charge profiles of $NiCo_2O_4$@PP cathode at different cycles.

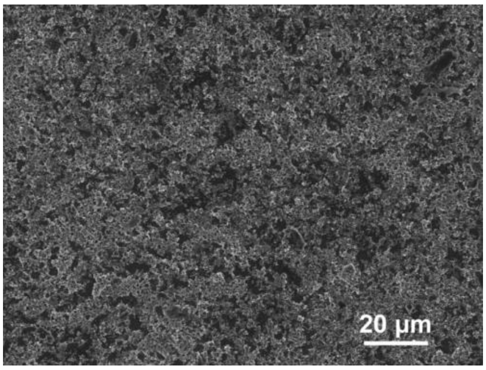

Figure S7. The surface SEM of composite separator after cyclic test.
